# Supplementary material for: Ezh2 is essential for the generation of functional yolk sac derived erythro-myeloid progenitors
Source: Nat Commun. 2021 Dec 2;12:7019. doi: 10.1038/s41467-021-27140-8 (PMC8640066; doi:10.1038/s41467-021-27140-8)
Supplement: Supplementary file 1 — Supplementary Information [file 41467_2021_27140_MOESM1_ESM.pdf]

Supplementary Information for:

Ezh2 is essential for the generation of functional yolk sac derived erythro-myeloid progenitors

Wen Hao Neo[1,2], Yiran Meng[3], Alba Rodriguez-Meira[1], Muhammad Z. H. Fadlullah[2], Christopher A. G. Booth[1], Emanuele Azzoni[3,4], Supat Thongjuea[5], Marella F. T. R. de Bruijn[3], Sten Eirik W. Jacobsen[1,6], Adam J. Mead[1*] and Georges Lacaud[2*]

Correspondence: wenhao.neo@cruk.manchester.ac.uk (W.H.N), adam.mead@imm.ox.ac.uk (A.J.M) and georges.lacaud@cruk.manchester.ac.uk (G.L)

1. Supplementary Figures

Supplementary Fig. 1. Related to Fig. 1
Supplementary Fig. 2. Related to Fig. 1
Supplementary Fig. 3. Related to Fig. 1
Supplementary Fig. 4. Related to Fig. 2
Supplementary Fig. 5. Related to Fig. 2
Supplementary Fig. 6. Related to Fig. 2
Supplementary Fig. 7. Related to Fig. 3
Supplementary Fig. 8. Related to Fig. 4
Supplementary Fig. 9. Related to Fig. 4

2. Supplementary Tables

Supplementary Table 1. Mouse genotyping PCR primers.

Supplementary Table 2. Antibodies used for FACS analysis and sorting experiments.

Supplementary Table 3. Antibodies used for immunofluorescence staining.

Supplementary Table 4. Pharmacological drugs.

Supplementary Table 5. Antibodies used for CUT&RUN.

Supplementary Table 6. Taqman probes.

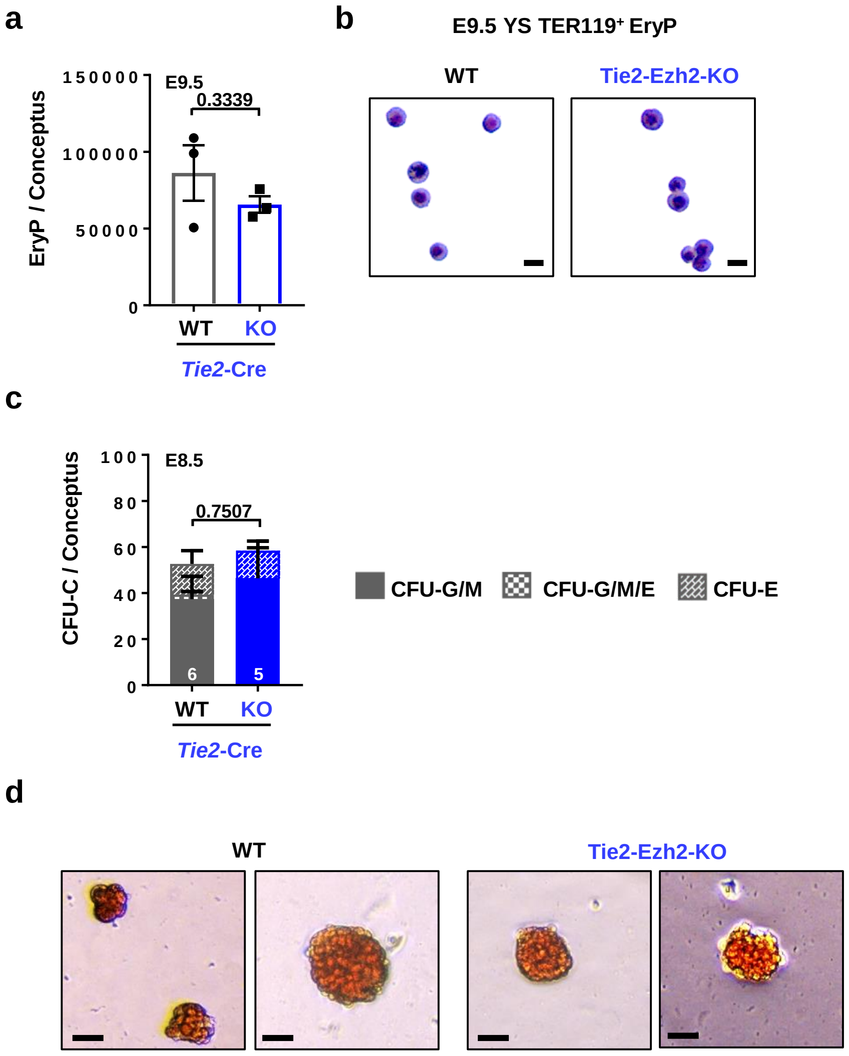

Supplementary Fig. 1 Normal primitive hematopoiesis in Tie2-Ezh2-KO embryo. **a** Total number of primitive erythroid (EryP) in E9.5 embryos (n = 3, 2 independent experiments). **b** Representative images of Wright-Giemsa stained cytospins from E9.5 TER119$^+$ EryP. Scale bars, 20 µm. **c** Number of CFU-C in E8.5 concepti (the head was removed for genotyping). The numbers of biologically independent replicates are indicated at the bottom of each column (2 independent experiments). **d** Representative images of EryP colonies from E8.5 CFU-C assay (2 independent experiments). Scale bars, 40 µm. Two-tailed t-test was used to assess statistical significance in **a**. 2way ANOVA was used to assess statistical significance in **c**. Error bars represent ± or + SEM.

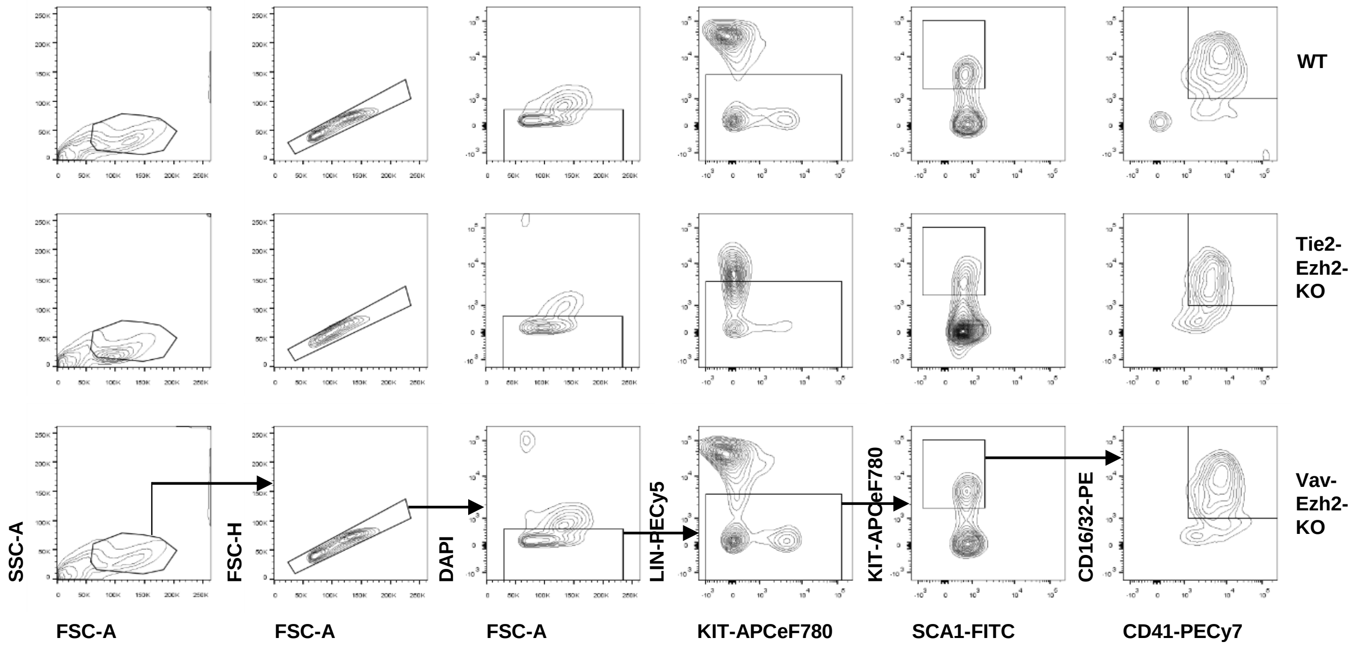

Supplementary Fig. 2 EMP FACS analysis.

Gating strategy used to define E10.5 YS EMP population.

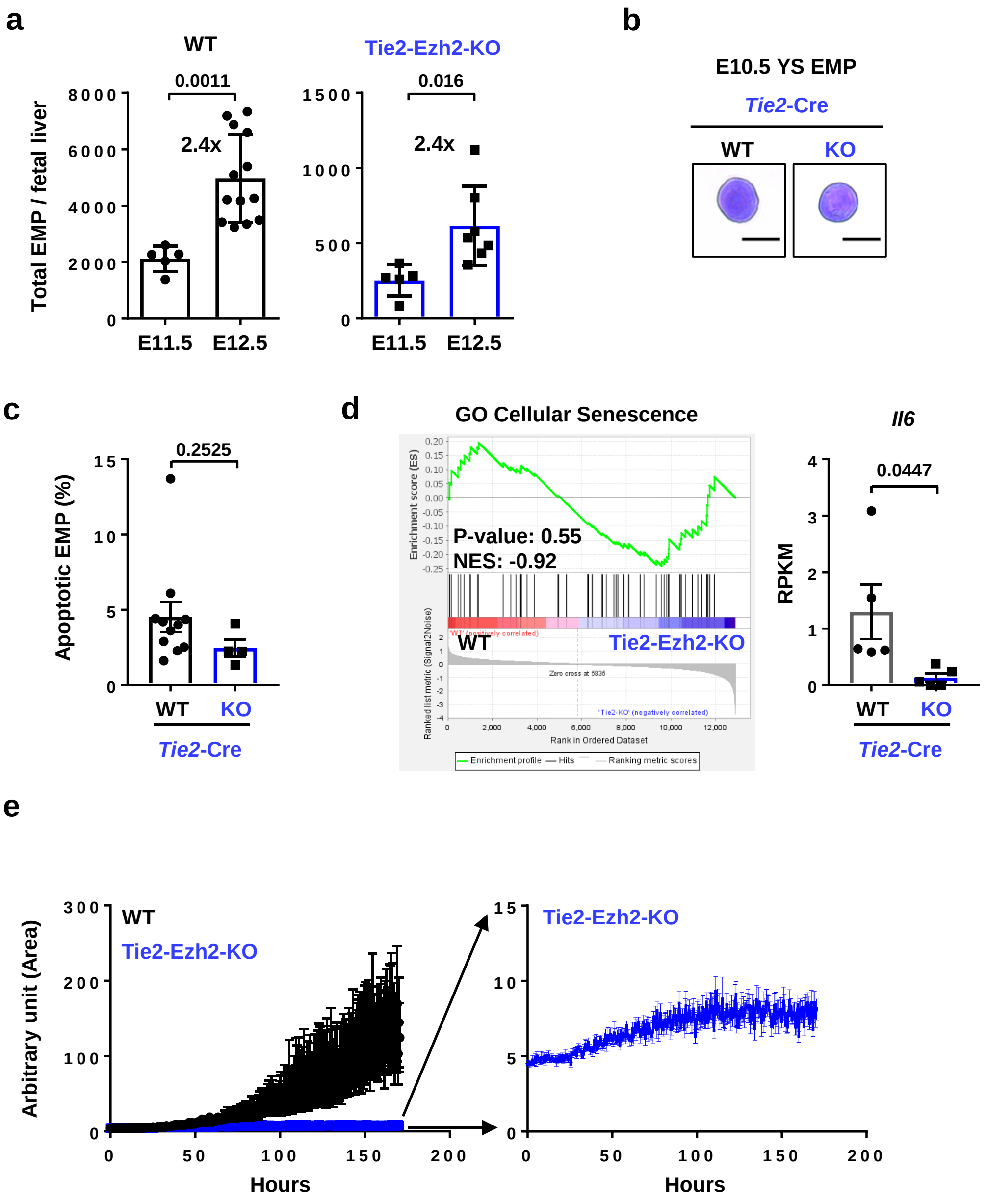

Supplementary Fig. 3 Phenotyping Tie2-Ezh2-KO EMPs. **a** Total number of EMPs in E11.5 and E12.5 fetal liver. Fold of increment between E10.5 and E11.5 was indicated (WT, E11.5, n = 5; WT, E12.5, n = 13; Tie2-Ezh2-KO, E11.5, n = 5; Tie2-Ezh2-KO, E12.5, n = 7; 3 independent experiments). **b** Representative images of Wright-Giemsa stained cytospins from E10.5 YS EMPs (2 independent experiments). Scale bars, 20 µm. **c** Percentage of apoptotic (Annexin $V^{+}7AAD^{-}$) EMP in E10.5 YS (Tie2-Ezh2-WT, n = 11; Tie2-Ezh2-KO, n = 4; 2 independent experiments). **d** Left: GSEA comparing WT and Tie2-Ezh2-KO EMPs for GO cellular senescence gene sets. Right: Expression of *Il6* in WT and Tie2-Ezh2-KO EMPs. Vav-Ezh2-WT (n = 2), Tie2-Ezh2-WT (n = 3) and Tie2-Ezh2-KO (n = 5). Each replicate represents 100 purified EMPs from individual YS at E10.5 (3 independent experiments). **e** Growth curves of E10.5 YS EMPs in liquid culture. Zoomed in section of Tie2-Ezh2-KO EMPs growth curve on the right. Two-tailed t-test was used to assess statistical significance in **a, c, d**. Error bars represent ± SEM.

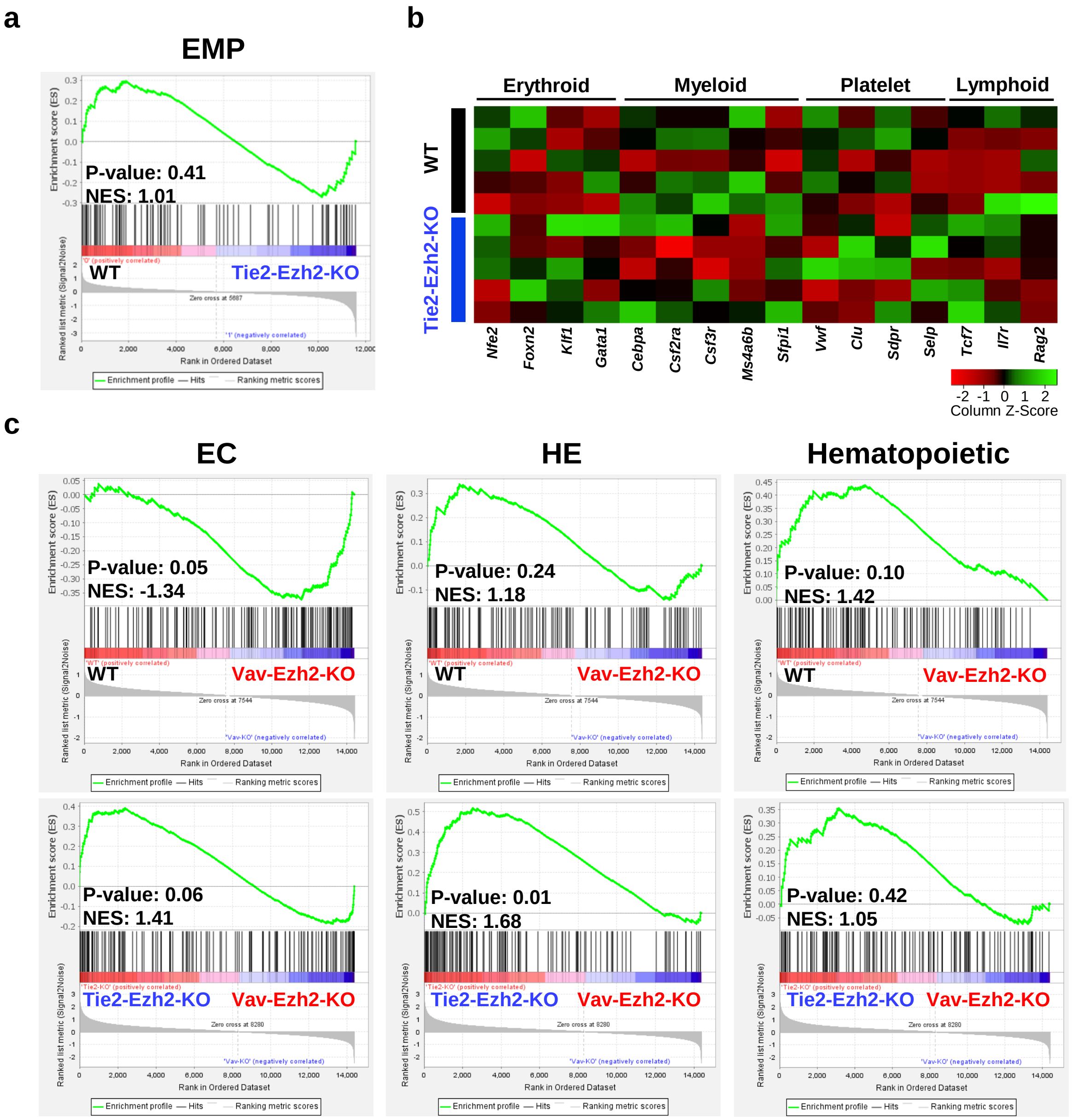

Supplementary Fig. 4 a GSEA comparing WT and Tie2-Ezh2-KO EMPs for EMP gene sets. **b** Heatmap for expression of lineage-affiliated genes in WT and Tie2-Ezh2-KO EMPs. **c** GSEA comparing WT, Vav-Ezh2-KO and Tie2-Ezh2-KO EMPs for EC, HE, hematopoietic specific gene sets. NES, normalized enrichment score.

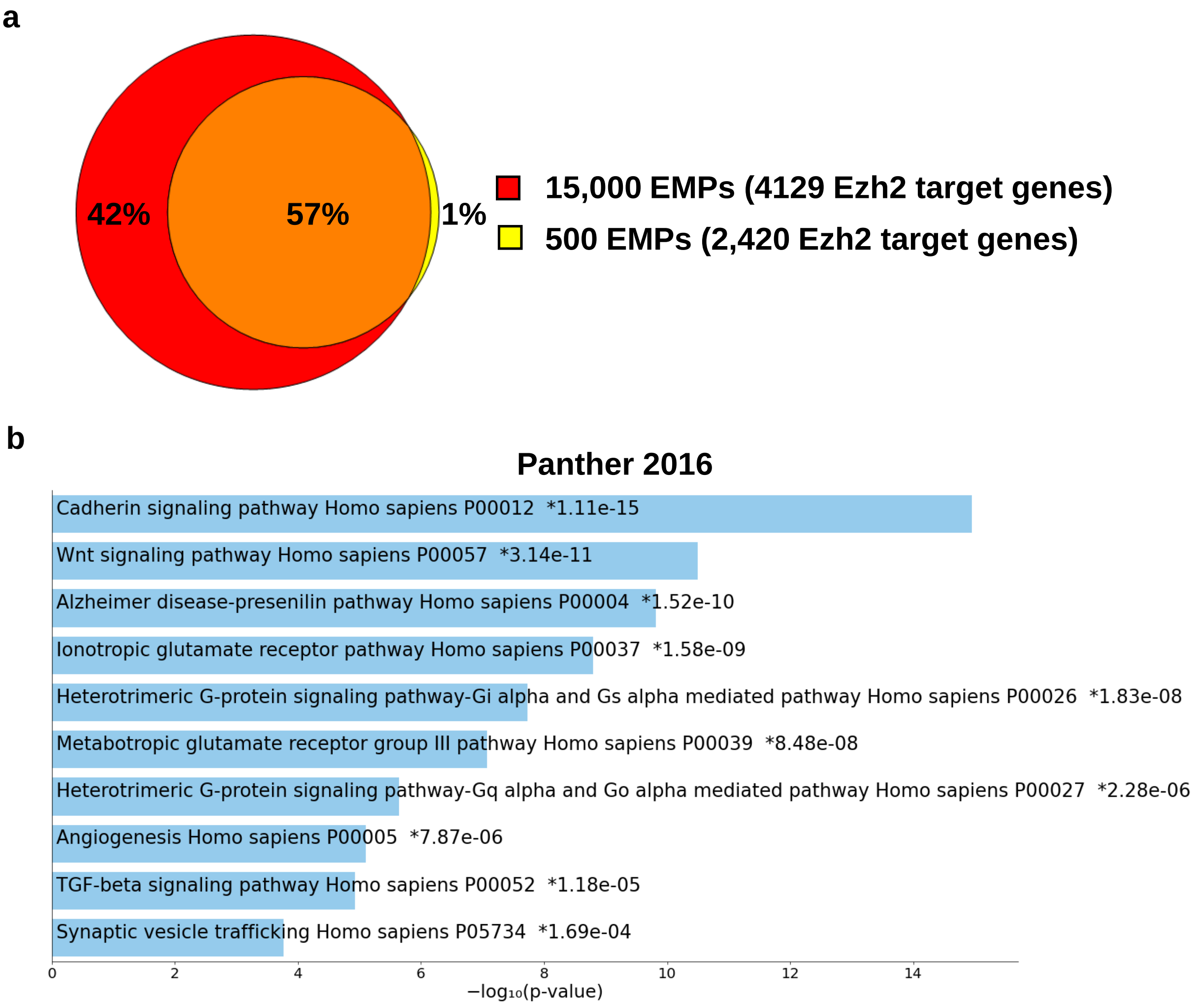

Supplementary Fig. 5 a Overlap of H3K27me3 positive genes (Ezh2 target genes) profiled by CUT&RUN with 500 or 15,000 E10.5 YS EMPs. 5,641 peaks which corresponded to 2,420 potential Ezh2 target genes (500 EMPs; ±3kb from TSS; $P < 0.01$) and 7,611 peaks which corresponded to 4,129 potential Ezh2 target genes (15,000 EMPs; ±3kb from TSS; $P < 0.05$) were detected. **b** Gene ontology enrichment analysis on Ezh2 target gene list with Panther 2016 using Enrichr. P values are computed from the Fisher exact test.

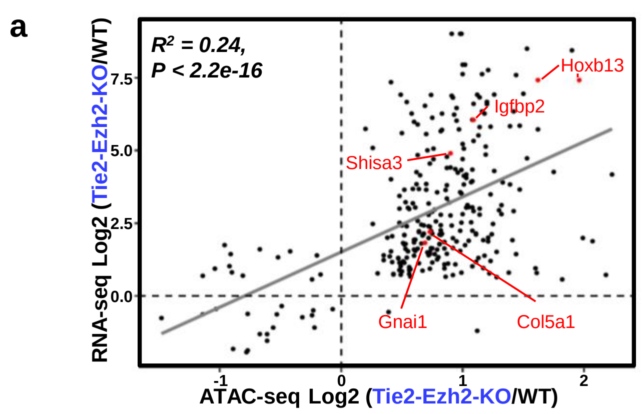

Supplementary Fig. 6 a Scatterplot depicting a significant positive correlation between RNA-seq (selected DE genes, P < 0.05, RPKM > 0.2) and ATAC-seq (P < 0.05) dataset. Statistical significance was determined based on the Pearson correlation coefficients. **b** Expression of Ezh2 target, hematopoietic-, EC-, HE- and Wnt signalling-associated genes in WT, Vav-Ezh2-KO and Tie2-Ezh2-KO EMPs. Vav-Ezh2-WT (n = 2), Tie2-Ezh2-WT (n = 3), Vav-Ezh2-KO (n = 5) and Tie2-Ezh2-KO (n = 5). Each replicate represents 100 purified EMPs from individual YS at E10.5 (3 independent experiments). Error bars represent ± SEM. Two-tailed t-test were used to assess statistical significance. **c** ATAC-seq enrichment at individual gene locus shown in **b**. Vav-Ezh2-WT (n = 3), Tie2-Ezh2-WT (n = 3), Vav-Ezh2-KO (n = 5) and Tie2-Ezh2-KO (n = 3). Each replicate represents 500 purified EMPs from individual YS at E10.5 (2 independent experiments). **d** IgG and H3K27me3 CUT&RUN tracks at individual gene locus shown in **b** (15,000 EMPs, 1 experiment).

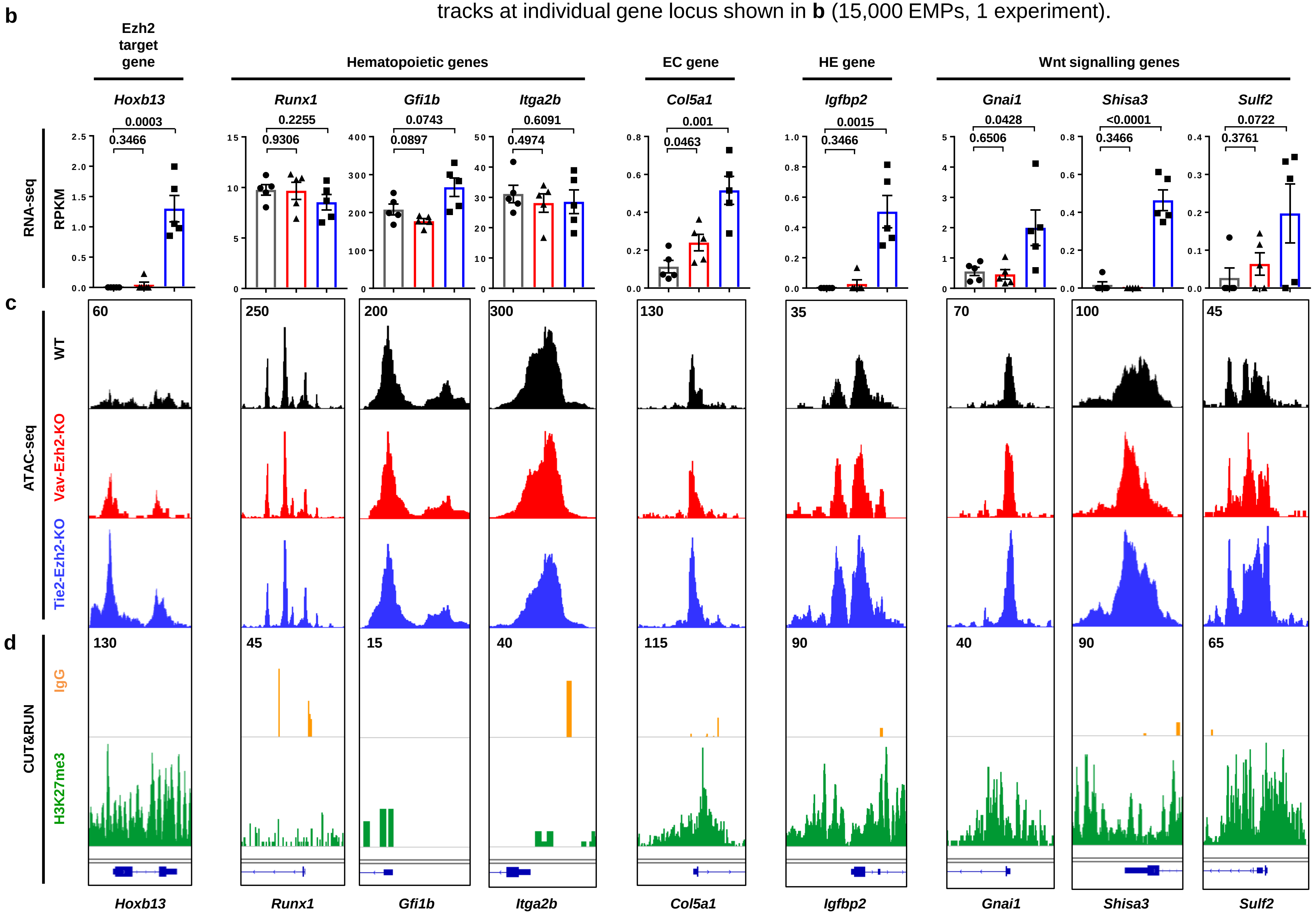

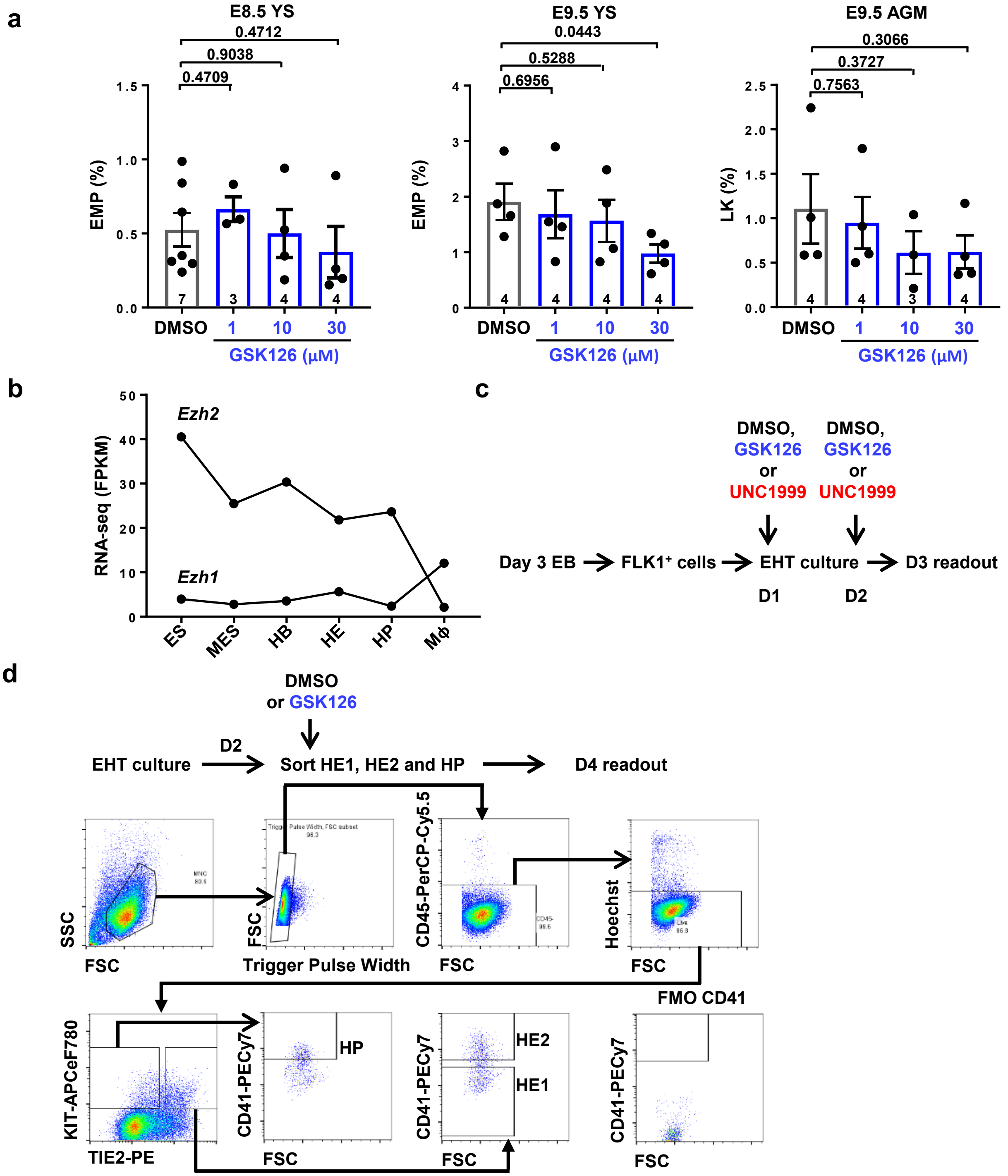

Supplementary Fig. 7 a Percentage (%) of YS EMP per YS and AGM LK (LIN⁻KIT⁺) per AGM treated with an increasing dosage of GSK126 (2 independent experiments). The numbers of biologically replicates are indicated at the bottom of each column. **b** *Ezh2* and *Ezh1* expression determined by RNA-seq across six stages of differentiation. FPKM, fragments/kilobase of transcript/million mapped reads. **c** Scheme of the experimental setup for **Fig. 3b-e**. mESCs were differentiated to EBs before FLK1⁺ cells isolation. EZH2 (GSK126) or EZH2/EZH1 (UNC1999) inhibition was started at day 1 (D1) or day 2 (D2) in EHT culture. Cultures were analysed at day 3 (D3) by FACS and CFU assays. **d** Scheme of the experimental setup for **Fig. 3f** and gating strategy used to define HE1, HE2 and HP. HE1, HE2 and HP were sorted from D2 EHT culture and treated with DMSO or GSK126 for two days before harvested for CFU-C assay. Two-tailed t-test was used to assess statistical significance. Error bars represent ± SEM.

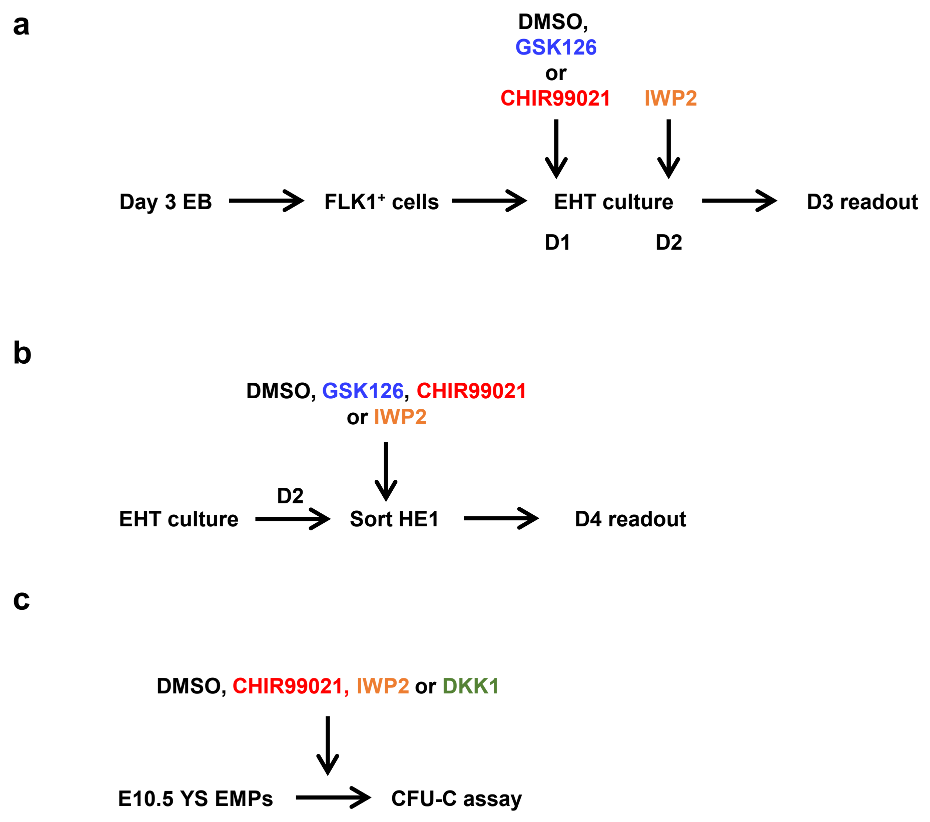

Supplementary Fig. 8 **a** Scheme of the experimental setup for **Fig. 4d, e**. mESCs were differentiated to EBs before isolation of FLK1$^+$ cells. Cultures were treated with or without GSK126 at day 1 (D1) and CHIR99021 or IWP2 at day 2 (D2). Cultures were analysed at day 3 (D3) by FACS and CFU-C assays. **b** Scheme of the experimental setup for **Fig. 4f**. HE1 were sorted from D2 EHT culture and treated with or without GSK126, CHIR99021 or IWP2 for two days before harvested for CFU-C assay. **c** Scheme of the experiment setup for **Fig. 4g**. E10.5 YS EMPs CFU-C assay with either DMSO, CHIR99021, IWP2 or DKK1. YS were dissociated and analysed at D3 by FACS and EMPs were sorted for CFU-C assays.

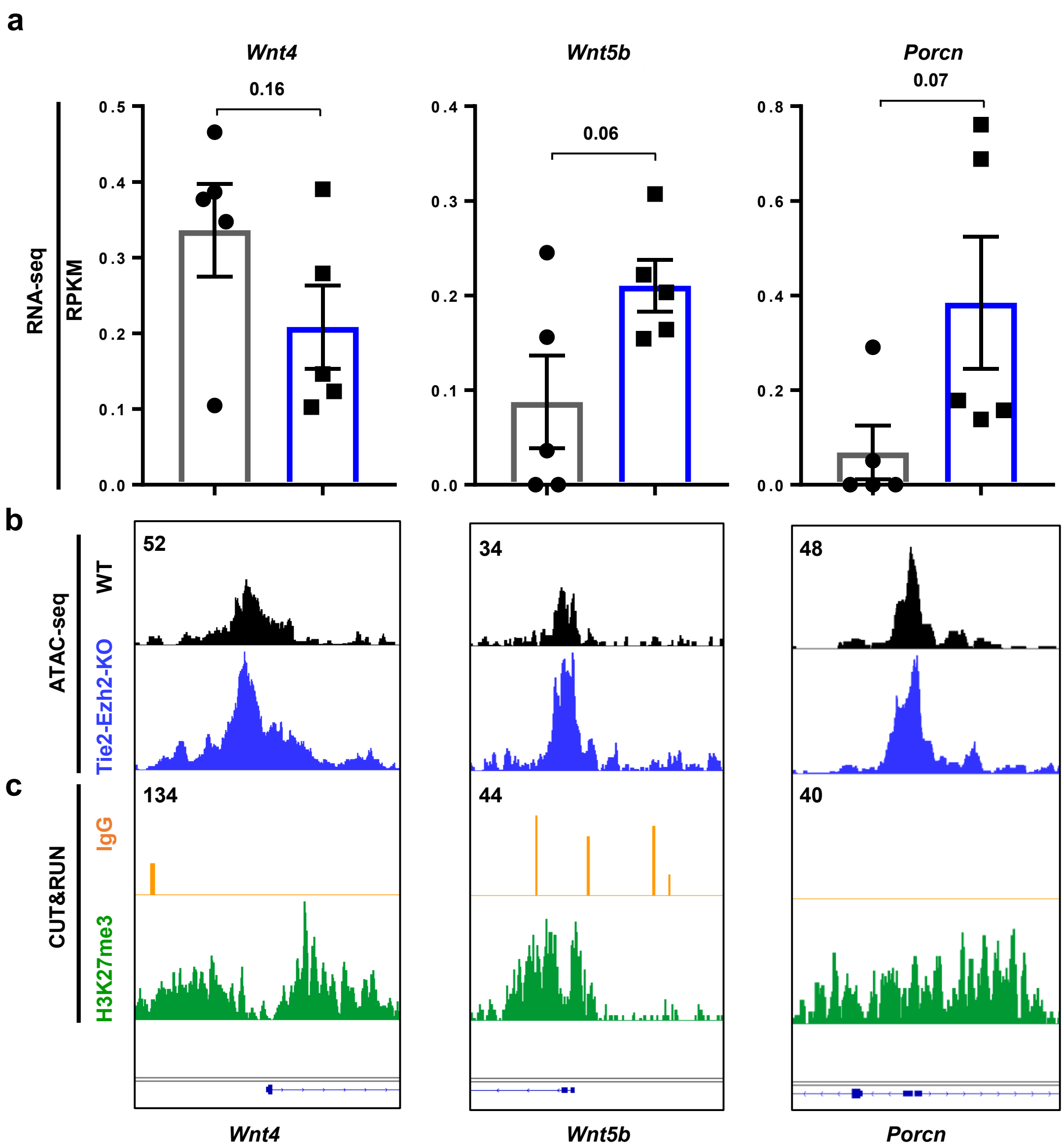

Supplementary Fig. 9 a Expression of *Wnt4*, *Wnt5b* and *Porcn* in WT and Tie2-Ezh2-KO EMPs. Vav-Ezh2-WT (n = 2), Tie2-Ezh2-WT (n = 3) and Tie2-Ezh2-KO (n = 5). Each replicate represents 100 purified EMPs from individual YS at E10.5 (3 independent experiments). Error bars represent ± SEM. Two-tailed t-test were used to assess statistical significance. **b** ATAC-seq enrichment at individual gene locus shown in **a**. Vav-Ezh2-WT (n = 3), Tie2-Ezh2-WT (n = 3), Vav-Ezh2-KO (n = 5) and Tie2-Ezh2-KO (n = 3). Each replicate represents 500 purified EMPs from individual YS at E10.5 (2 independent experiments). **c** IgG and H3K27me3 CUT&RUN tracks at individual gene locus shown in **a** (15,000 EMPs, 1 experiment).

Supplementary Table 1. Mouse genotyping PCR primers.

Mouse line	Primers 5' - 3'	Product
Ezh2	F: CTGCTCTGAATGGCAACTCC<br>R: TTATTCATAGAGCCACCTGG	WT: 430 bp<br>Floxed: 470 bp
Tie2-Cre	F: CGTTTTCTGAGCATACCTGGA<br>R: ATTCTCCCACCGTCAGTACG	Tg: 450 bp
Vav-iCre	F: AGATGCCAGGACATCAGGAACCTG<br>R: ATCAGCCACACCAGACACAGAGATC	Tg: 250 bp
Rosa26-LSL-tdTomato	WT F: AAGGGAGCTGCAGTGGAGTA<br>WT R: CCGAAAATCTGTGGGAAGTC<br>KI F: GGCATTAAAGCAGCGTATCC<br>KI R: CTGTTCCTGTACGGCATGG	WT: 297 bp<br>KI: 196 bp

Supplementary Table 2. Antibodies used for FACS analysis and sorting experiments.

Antibody	Clone	Manufacturer	Cat. number	Dilution
CD3e PECy5	145-2C11	Biolegend	100310	1:100
B220 PECy5	RA3-6B2	Biolegend	103210	1:400
F4/80 PECy5	BM8	Biolegend	123112	1:200
TER119 PECy5	TER-119	Biolegend	116210	1:600
GR1 PECy5	RB6-8C5	Biolegend	108410	1:800
IL-7R PECy5	A7R34	eBioscience	15-1271-83	1:200
SCA1 PB	E13-161.7	Biolegend	122520	1:200
CD16/CD32 PE	93	eBioscience	12-0161-83	1:400
KIT APC eF780	2B8	eBioscience	47-1171-82	1:1600
CD41a PECy-7	MWReg30	eBioscience	25-0411-82	1:800
CD45 PE-Texas Red	30-F11	Invitrogen	MCD4517	1:100
CD144 APC	eBioBV13	eBioscience	17-1441-82	1:200
TIE2 PE	TEK4	eBioscience	12-5987-83	1:200
Annexin V APC		Invitrogen	A35110	1:20
Hoechst 33258		Invitrogen	H3569	1:50
7-amino-actinomycin D (7-AAD)		Sigma	SML1633-1ML	1:100
DAPI		Invitrogen	62248	1:1000

Supplementary Table 3. Antibodies used for immunofluorescence staining.

Antibody	Clone	Manufacturer	Cat. number	Concentration/Dilution
CD31	390	R&D Systems	AF3628	0.2 µg/ml
RUNX1	EPR3099	Abcam	ab92336	1:200
KIT	2B8	eBioscience	14-1171	2.0 µg/ml
β-CATENIN	D2U8Y	Cell Signaling Technologies	19807S	1:200
Alexa Fluor 647 chicken anti rat		Thermo Fisher	A-21472	1:400
Alexa Fluor 555 donkey anti goat		Thermo Fisher	A-21432	1:400
Alexa Fluor 488 donkey anti rabbit		Thermo Fisher	A-21208	1:400
Alexa Fluor 555 donkey anti rabbit		Thermo Fisher	A-32794	1:400

Supplementary Table 4. Pharmacological drugs.

Drugs	Manufacturer	Cat. number
GSK126	LKT Laboratories	G7340
UNC1999	APExBIO	B1583
IWP2	Adooq Bioscience	A12707-10
DKK1	R&D Systems	5897-DK-010
CHIR99021	Adooq Bioscience	A10199-5

Supplementary Table 5. Antibodies used for CUT&RUN.

Antibody	Clone	Manufacturer	Cat. number	Concentration/Dilution
Rabbit mAb IgG XP® isotype control	DA1E	Cell Signaling Technologies	66362	5µl/sample
H3K27me3		Merck MilliPore	07-449	1:100

Supplementary Table 6. Taqman probes.

Gene name	Probe ID
Ezh2	Mm00468464_m1
Hprt	Mm01545399_m1
