# Supplementary material for: Ezh2 is essential for the generation of functional yolk sac derived erythro-myeloid progenitors
Source: Nat Commun. 2021 Dec 2;12:7019. doi: 10.1038/s41467-021-27140-8 (PMC8640066; doi:10.1038/s41467-021-27140-8)
Supplement: Supplementary file 3 — Description of Additional Supplementary Files [file 41467_2021_27140_MOESM3_ESM.pdf]

Description of Additional Supplementary Files

File Name: Supplementary Data 1
Description: RNA-seq Data for WT versus Tie2-Ezh2-KO EMP (RPKMs, Fold Changes and Significance).

File Name: Supplementary Data 2
Description: H3K27me3 CUT&RUN.

File Name: Supplementary Data 3
Description: Hallmark GSEA for Tie2-Ezh2-KO EMP.

File Name: Supplementary Data 4
Description: Gene signatures for GSEA.

File Name: Supplementary Movie 1
Description: Time-lapse imaging of WT E10.5 YS EMPs.

File Name: Supplementary Movie 2
Description: Time-lapse imaging of Tie2-Ezh2-KO E10.5 YS EMPs.

File Name: Supplementary Movie 3
Description: Time-lapse imaging of FLK1+ cells blast monolayer cultures treated with DMSO at day 1 (DMSO Ctrl).

File Name: Supplementary Movie 4
Description: Time-lapse imaging of FLK1+ cells blast monolayer cultures treated with GSK126 at day 1 (GSK126).
