# Supplementary material for: Ezh2 is essential for the generation of functional yolk sac derived erythro-myeloid progenitors
Source: Nat Commun. 2021 Dec 2;12:7019. doi: 10.1038/s41467-021-27140-8 (PMC8640066; doi:10.1038/s41467-021-27140-8)
Supplement: Supplementary file 12 — Reporting Summary [file 41467_2021_27140_MOESM12_ESM.pdf]

nature research

Corresponding author(s): Wen Hao Neo, Adam J. Mead, Georges Lacaud

Last updated by author(s): Oct 25, 2021

Reporting Summary

Nature Research wishes to improve the reproducibility of the work that we publish. This form provides structure for consistency and transparency in reporting. For further information on Nature Research policies, see our Editorial Policies and the Editorial Policy Checklist.

Statistics

For all statistical analyses, confirm that the following items are present in the figure legend, table legend, main text, or Methods section.

n/a	Confirmed	
☐	☒	The exact sample size (*n*) for each experimental group/condition, given as a discrete number and unit of measurement
☐	☒	A statement on whether measurements were taken from distinct samples or whether the same sample was measured repeatedly
☐	☒	The statistical test(s) used AND whether they are one- or two-sided<br>*Only common tests should be described solely by name; describe more complex techniques in the Methods section.*
☒	☐	A description of all covariates tested
☒	☐	A description of any assumptions or corrections, such as tests of normality and adjustment for multiple comparisons
☐	☒	A full description of the statistical parameters including central tendency (e.g. means) or other basic estimates (e.g. regression coefficient) AND variation (e.g. standard deviation) or associated estimates of uncertainty (e.g. confidence intervals)
☐	☒	For null hypothesis testing, the test statistic (e.g. *F*, *t*, *r*) with confidence intervals, effect sizes, degrees of freedom and *P* value noted<br>*Give P values as exact values whenever suitable.*
☒	☐	For Bayesian analysis, information on the choice of priors and Markov chain Monte Carlo settings
☒	☐	For hierarchical and complex designs, identification of the appropriate level for tests and full reporting of outcomes
☒	☐	Estimates of effect sizes (e.g. Cohen's *d*, Pearson's *r*), indicating how they were calculated

Our web collection on statistics for biologists contains articles on many of the points above.

Software and code

Policy information about availability of computer code

Data collection	Flow Cytometry data: BD FACSDiva 8.0.1.
Data analysis	Flow Cytometry data analysis: FlowJo 10.3<br>Data visualization and statistical comparison with t-test and ANOVA: GraphPad Prism 7.0<br>High throughput sequencing analysis (RNA-seq, CUT & RUN, ATAC-seq): R (v4.1.0), TopHat (v2.0.13), DESeq2 (1.32.0), GSEA (v2.2.0), Bowtie2 (v2.2.1), MACS (v2.1.2), ChIPpeakAnno (v3.20.1), deeptools (v3.5.1), SAMtools (v1.3.1), Picard (v1.96), Homer (v4.10), diffReps (v1.55.4).

For manuscripts utilizing custom algorithms or software that are central to the research but not yet described in published literature, software must be made available to editors and reviewers. We strongly encourage code deposition in a community repository (e.g. GitHub). See the Nature Research guidelines for submitting code & software for further information.

Data

Policy information about availability of data

All manuscripts must include a data availability statement. This statement should provide the following information, where applicable:
- Accession codes, unique identifiers, or web links for publicly available datasets
- A list of figures that have associated raw data
- A description of any restrictions on data availability

The RNA-seq, ATAC-seq and CUT&RUN data generated in this study have been deposited in the GEO database under accession code GSE181873 [https://www.ncbi.nlm.nih.gov/geo/query/acc.cgi?acc=GSE181873]. The mouse reference genome assembly GRCm38 was downloaded from Ensembl website http://ftp.ensembl.org/. Source data are provided with this paper.

Field-specific reporting

Please select the one below that is the best fit for your research. If you are not sure, read the appropriate sections before making your selection.

- [x] Life sciences
- [ ] Behavioural & social sciences
- [ ] Ecological, evolutionary & environmental sciences

For a reference copy of the document with all sections, see nature.com/documents/nr-reporting-summary-flat.pdf

Life sciences study design

All studies must disclose on these points even when the disclosure is negative.

Sample size	No statistical methods were used to predetermine the experimental sample size. Sample sizes were chosen based on our experience and commonly accepted sample sizes for similar experiments and analyses in literature. Sample sizes for all data sets are clearly listed in Methods and Figure legends.
Data exclusions	No data were excluded during the analysis.
Replication	Three independent experiments were performed for most experiments unless stated in the figure legend. All experiments consist more than three biological replicates. All attempts at replication were successful.
Randomization	Samples were allocated in different groups depending on their genotype and embryonic stages.
Blinding	Blinding was not performed, consistent with widespread practice in the field for studies of this nature.

Reporting for specific materials, systems and methods

We require information from authors about some types of materials, experimental systems and methods used in many studies. Here, indicate whether each material, system or method listed is relevant to your study. If you are not sure if a list item applies to your research, read the appropriate section before selecting a response.

Materials & experimental systems

n/a	Involved in the study	
[ ]	[x]	Antibodies
[ ]	[x]	Eukaryotic cell lines
[x]	[ ]	Palaeontology and archaeology
[ ]	[x]	Animals and other organisms
[x]	[ ]	Human research participants
[x]	[ ]	Clinical data
[x]	[ ]	Dual use research of concern

Methods

n/a	Involved in the study	
[ ]	[x]	ChIP-seq
[ ]	[x]	Flow cytometry
[x]	[ ]	MRI-based neuroimaging

Antibodies

Antibodies used

All antibodies used in the study were obtained from commercial vendors.
CD3e PECy5 145-2C11 Biolegend 100310 1:100
B220 PECy5 RA3-6B2 Biolegend 103210 1:400
F4/80 PECy5 BM8 Biolegend 123112 1:200
TER119 PECy5 TER-119 Biolegend 116210 1:600
GR1 PECy5 RB6-8C5 Biolegend 108410 1:800
IL-7R PECy5 A7R34 eBioscience 15-1271-83 1:200
SCA1 PB E13-161.7 Biolegend 122520 1:200
CD16/CD32 PE 93 eBioscience 12-0161-83 1:400
KIT APC eF780 2B8 eBioscience 47-1171-82 1:1600
CD41a PECy-7 MWReg30 eBioscience 25-0411-82 1:800
CD45 PE-Texas Red 30-F11 Invitrogen MCD4517 1:100
CD144 APC eBioBV13 eBioscience 17-1441-82 1:200
TIE2 PE TEK4 eBioscience 12-5987-83 1:200
Annexin V APC Invitrogen A35110 1:20
Hoechst 33258 Invitrogen H3569 1:50
7-amino-actinomycin D (7-AAD) Sigma SML1633-1ML 1:100
DAPI Invitrogen 62248 1:1000
CD31 390 R&D Systems AF3628 0.2 μg/ml
RUNX1 EPR3099 Abcam ab92336 1:200

KIT 2B8 eBioscience 14-1171 2.0 µg/ml
β-CATENIN D2U8Y Cell Signaling Technologies 19807S 1:200
Alexa Fluor 647 chicken anti rat Thermo Fisher A-21472 1:400
Alexa Fluor 555 donkey anti goat Thermo Fisher A-21432 1:400
Alexa Fluor 488 donkey anti rabbit Thermo Fisher A-21208 1:400
Alexa Fluor 555 donkey anti rabbit Thermo Fisher A-32794 1:400
Rabbit mAb IgG XP® isotype control DA1E Cell Signaling Technologies 66362 5µl/sample
H3K27me3 Merck MilliPore 07-449 1:100

Validation

All antibodies were validated by their manufacturers for the application (flow cytometry) and species (mouse) used in this study. In addition, all antibodies used were individually titrated before their use to identify their optimal working concentration. In all the experiments, fluorescence-minus-one (FMO) were included.

Eukaryotic cell lines

Policy information about cell lines

Cell line source(s)

mESC was generated from Kyba ey al, Cell, 2002.

Authentication

Cell lines were not authenticated. However, mESC has the capacity to differentiate into hematopoietic cells with our in house protocol and it has been validated routinely.

Mycoplasma contamination

Mycoplasma contamination were tested by PCR and found negative in all routine tests.

Commonly misidentified lines
(See ICLAC register)

No commonly misidentified lines were used.

Animals and other organisms

Policy information about studies involving animals; ARRIVE guidelines recommended for reporting animal research

Laboratory animals

Ezh2fl/fl (59), Tie2-Cre (23), Vav-iCre (22) and Rosa26-LSL-tdTomato (60) reporter mice used in this study have been previously described. All mice were either on C57BL/6 (CD45.2) background or backcrossed to C57BL/6 (>5 generations). All mice were bred and maintained in accordance with UK Home Office project license 30/3103 and 70/8472. Embryonic development was estimated considering the day of vaginal plug formation as 0.5 d post-coitum (dpc) and somite pairs (sp). Both male and female mice were used. Genotyping primers are details in Supplementary Table 1. Mice were housed under specific-pathogen-free conditions with standard food and water ad libitum in a 12h light / 12h dark cycle. Humidity and ambient temperature were maintained between 45-65% and 20-24°C, respectively.

Wild animals

No wild animals were used in the study.

Field-collected samples

No field collected samples were used in the study.

Ethics oversight

All experimental procedures and mouse breeding and maintenance were in accordance with United Kingdom Home Office regulations. All experiments were approved by the Oxford University Clinical Medicine Ethical Review Committee and Cancer Research United Kingdom-Manchester Institute Animal Welfare and Ethical Review Body.

Note that full information on the approval of the study protocol must also be provided in the manuscript.

ChIP-seq

Data deposition

- [x] Confirm that both raw and final processed data have been deposited in a public database such as GEO.
- [x] Confirm that you have deposited or provided access to graph files (e.g. BED files) for the called peaks.

Data access links
May remain private before publication.

Data files have been uploaded to GEO: GSE181873.

Files in database submission

RNA-seq (GSE150032):
Tie2-Ezh2-WT4
Tie2-Ezh2-WT10
Tie2-Ezh2-WT12
Vav-Ezh2-WT4
Vav-Ezh2-WT6
Tie2-Ezh2-KO7
Tie2-Ezh2-KO9
Tie2-Ezh2-KO11
Tie2-Ezh2-KO13
Tie2-Ezh2-KO17

Vav-Ezh2-KO1
Vav-Ezh2-KO9
Vav-Ezh2-KO10
Vav-Ezh2-KO26
Vav-Ezh2-KO28
ATAC-seq (GSE150292):
Tie2-Ezh2-WT2
Tie2-Ezh2-WT3
Tie2-Ezh2-KO5
Tie2-Ezh2-KO7
Tie2-Ezh2-KO9
Tie2-Ezh2-KO10
Tie2-Ezh2-KO11
Tie2-Ezh2-WT12
Vav-Ezh2-KO1
Vav-Ezh2-KO13
Vav-Ezh2-KO18
Vav-Ezh2-WT2
Vav-Ezh2-WT16
Vav-Ezh2-WT17
CUT&RUN (GSE181869):
IgG 500 cell [CUT&RUN]
IgG 15000 cell [CUT&RUN]
H3K27me3 500 cell [CUT&RUN]
H3K27me3 15000 cell [CUT&RUN]

Genome browser session (e.g. UCSC)

GEO data is made publicly available. BigWig and BED files can be downloaded at GEO.

Methodology

Replicates

CUT&RUN were done in two independent experiments with 500 or 15,000 YS EMPs.

Sequencing depth

500 cell YS EMP; Sequencing depth: 8.6 Million, Alignment rate: 17%, Read length: 76bp, Paired-end
15,000 cell YS EMP; Sequencing depth: 19.1 Million, Alignment rate: 85%, Read length: 59bp, Paired-end

Antibodies

Antibodies used for CUT&RUN were Rabbit (DA1E) mAb IgG XP® isotype control (3900S, Cell Signaling Technologies) and anti-H3K27me3 (07-449, Merck MilliPore).

Peak calling parameters

Mapping parameter (bowtie2 version 2.2.1), reference genome:GRCm38
For all samples; (bowtie2 -p 4 --dovetail --phred33 -x ${GenomeIndex} -1 ${inputFastq_R1} -2 ${inputFastq_R2}) 2> ${logDir}/${samp}.bowtie2 | samtools view -bS - > ${outDir}/${samp}_aligned_reads.bam

Peak calling parameters (MACS2 version 2.1.2)
500 cell YS EMP; MACS2 callpeak -t ${H3k27me3_15000_cell.bam} -c ${IgG_15000_cell.bam} --format BAM -g mm --broad --pvalue 0.01 --broad-cutoff 0.01
15,000 cell YS EMP; MACS2 callpeak -t ${H3k27me3_500_cell.bam} -c ${IgG_500_cell.bam} --format BAM -g mm --broad --pvalue 0.05 --broad-cutoff 0.05

Data quality

We followed the ENCODE standard and recommended software settings for data processing, and visually verified at least 20 identified peaks in each sample.

Software

Peaks were annotated with the Bioconductor package ChIPpeakAnno (version 3.26.2).

Flow Cytometry

Plots

Confirm that:

- [x] The axis labels state the marker and fluorochrome used (e.g. CD4-FITC).
- [x] The axis scales are clearly visible. Include numbers along axes only for bottom left plot of group (a 'group' is an analysis of identical markers).
- [x] All plots are contour plots with outliers or pseudocolor plots.
- [x] A numerical value for number of cells or percentage (with statistics) is provided.

Methodology

Sample preparation	Cells were incubated in Fc-block before being stained with antibodies at predetermined (titrated) optimized concentrations. Gates were set using a combination of fluorescence-minus-one controls and also populations that are known to be negative for the antigen.
Instrument	Data collection were done with BD FACSAriaII/III, BD Aria Fusion, BD Influx, BD LSRII and LSRFortessa X-20.
Software	BD FACSDiva was used for data collection for all experiments. FlowJo 10.3 was used for data analysis.
Cell population abundance	EMP: 1%-2% of YS viable mononuclear cells<br>HE1: 1% of viable CD45-negative cells<br>HE2: 1.5% of viable CD45-negative cells<br>HP: 1% of viable CD45-negative cells
Gating strategy	FSC-A/SSC-A was used for gating mononuclear cells. FSC-A/FSC-H was used for gating on singlets. Viability dye was used to exclude non-viable cells. Where relavent, lineage-cocktail was used for gating out lineage-positive cells. Specific gating strategies are included in the Supplementary Figures.

☒ Tick this box to confirm that a figure exemplifying the gating strategy is provided in the Supplementary Information.
